# Multifactorial Contributors to the Longevity of Dental Restorations: An Integrated Review of Related Factors

**DOI:** 10.3390/dj12090291

**Published:** 2024-09-12

**Authors:** Maria Jacinta Moraes Coelho Santos, Elham Zare, Peter McDermott, Gildo Coelho Santos Junior

**Affiliations:** 1 Schulich School of Medicine and Dentistry, Western University London, London, ON N6A 3K7, Canada; jacinta.santos@schulich.uwo.ca (M.J.M.C.S.); peter.mcdermott@schulich.uwo.ca (P.M.); 2 Interdisciplinary Medical Science, Schulich School of Medicine and Dentistry, London, ON N6A 3K7, Canada; ezare@uwo.ca

**Keywords:** dental restorations, longevity, tooth-related factors, patient-related factors, dentist-related factors, restoration failure, direct restorations, amalgam, composite resin, glass ionomer cement

## Abstract

Purpose: This integrated review aims to identify and analyze the multifactorial contributors to the longevity of direct restorations, focusing on tooth-, patient-, and dentist-related factors. Materials and Methods: A search of the literature was performed using an electronic database, PubMed/Medline, Web of Science, and Scopus, on papers published between 1980 and 2024. The titles and abstracts of papers that evaluated aspects categorized into tooth-related, patient-related, and dentist-related factors influencing restoration failure were selected and screened. Full-text assessments were conducted, and the extracted data were compiled, summarized, and synthesized. The reference lists of the collected papers were also screened, and relevant citations were included in this review. Data were gathered from clinical and laboratorial studies, systematic reviews, and meta-analyses to provide a comprehensive understanding of restoration longevity. Results: Among the tooth-related factors, multiple-surface restorations, deep margins, tooth location, and tooth vitality significantly impact restoration survival. Patient-related factors such as medical conditions, risk predictors of caries, age, sex, parafunctional habits, smoking, periodontal health, number of restorations, and socioeconomic status all play crucial roles. Regarding dentist-related factors, the decision-making process, age, experience, and dentist manual dexterity are vital aspects. Furthermore, the technique used, including isolation methods for moisture control, as well as the type of dental practice (large group vs. small practice), notably influenced the restoration survival. Conclusions: The longevity of dental restorations is influenced by a complex interplay of tooth-related, patient-related, and dentist-related factors. Strategies to improve restoration outcomes should consider all these multifactorial contributors. Continuing professional education, diligent patient guidance on the factors that influence restoration survival, careful material selection and restorative technique, and tailored individual treatment are crucial factors to reduce failure rates and improve the lifespan of restorations.

## 1. Introduction

Direct restorations are widely used to treat dental caries lesions due to several factors, including their reduced treatment complexity, cost-effectiveness, decreased chair time, and fewer clinical sessions. The primary materials employed for direct restorations are composite resin, amalgam, resin-modified glass ionomer (RMGI), and glass ionomer cement (GIC) [1]. Despite their widespread use, the longevity of direct restorations varies, with approximately half of all restorations placed in general dental practice being replacements for defective or failed restorations [2,3,4,5]. This leads to significant costs, with global expenses estimated at USD 298 billion yearly, corresponding to an average of 4.6% of global health expenditure [6]. Furthermore, it is crucial to account for indirect costs, primarily those associated with productivity losses due to work absences, which account for an additional USD 144 billion. The annual failure rate for amalgam restorations ranges from 0.16% to 2.83% [7], while for composite restorations, it varies between 1% and 4% [8,9,10]. Research indicates that dental students can attain satisfactory clinical outcomes using posterior composites, with an average yearly failure rate of 2.8% [11]. Indeed, strict adherence to adhesive protocols and the proper placement of direct composite restorations can result in them lasting for up to three decades, demonstrating a clinically satisfactory performance [12]. GIC restorations, on the other hand, exhibit an annual failure rate of 7%, mainly due to deficiencies in the material [13,14,15,16,17].

The failure of restorations imposes a burden on both the healthcare system and patients due to additional financial commitments and time resources. Furthermore, the frequent replacement of restorations can lead to additional trauma to the tooth, including further reductions in dental tissue, loss of tooth vitality, and reduced patient trust [18,19,20,21,22]. Studies have identified moderately strong evidence indicating a genetic contribution to caries susceptibility, particularly involving genes related to enamel formation, salivary properties, and immune regulation [23,24]. However, the genetic risk is further modified by lifestyle and environmental factors. Patients’ lifestyle, behavior, dietary habits, and systemic diseases have been proven to influence the development of dental caries and periodontal diseases [23]. Fermentable carbohydrates are the most significant dietary risk factor for caries disease. Their breakdown results in acid production and biofilm formation, which lowers pH and favors acid-producing microorganisms. The concentration, availability, and adhesiveness of these carbohydrates in food also contribute significantly [23,25].

The most frequent causes of failure for direct restorations may vary slightly according to the restorative material. For amalgam restorations, these include secondary caries, tooth/restoration fracture, marginal deficiencies, restoration dislodgement, and overhang [26]. The main reasons for composite resin restoration failure include secondary caries, tooth/restoration fracture, use of a thick layer of glass ionomer liner, the absence of peripheral enamel bonding, and enamel beveling deficiency [27]. For GIC/RMGI restorations, the primary factors for failure are secondary caries and the loss of glass-ionomer material [28].

Understanding the factors that influence the success and failure of dental restorations is essential for improving clinical outcomes, decreasing the need for repairs or replacements, and reducing the financial burden on healthcare systems. To enhance the success rates of direct restorations, it is vital to identify the causes of early restoration failure and develop strategies to mitigate these failures. This study aims to analyze the multifactorial contributors to the longevity of dental restorations through an integrated review of tooth-related, patient-related, and dentist-related factors.

## 2. Materials and Methods

A search of the literature was performed using an electronic database, PubMed/Medline, Web of Science, and Scopus, including papers published between 1980 and 2024. The titles and abstracts of papers that evaluated aspects categorized into tooth-related, patient-related, and dentist-related factors influencing restoration failure were selected and screened. Full-text assessments were conducted independently by two reviewers and in the presence of disagreements, a resolution was reached through careful analysis and discussion involving a third author. The reference lists of the collected papers were also screened, and relevant citations were included in this review. Data were gathered from clinical and laboratorial studies, systematic reviews, and meta-analyses to provide a comprehensive understanding of restoration longevity. Inclusion criteria: English language; prospective and retrospective clinical trials; laboratorial studies; meta-analyses; systematic review; and a literature review. Exclusion criteria: papers published before 1980; studies conducted in primary teeth; in vitro studies conducted on bovine teeth; duplicated studies; and studies considered to have a high risk of bias. Any disagreements regarding the quality rating of individual studies were resolved through a consensus among the reviewers.

## 3. Results

The initial database search identified 798 titles. After duplicates were removed and titles were analyzed, 296 abstracts were screened. A total of 242 papers underwent thorough examination, and additional papers were excluded for not meeting the eligibility criteria. Moreover, an additional 33 papers were included after assessing the original data. Ultimately, 172 studies were included in this integrated review, focusing on the multifactorial contributors to the longevity of dental restorations. The results were categorized into three main factors: patient-related, dentist-related, and tooth-related factors. Table 1, Table 2 and Table 3 summarize the main findings from this comprehensive review.

## 4. Discussion

### 4.1. Patient-Related Factors

Behavioral factors of an individual, such as snacking frequency and toothbrushing habits, can directly affect oral environmental conditions, which in turn influence the survival of restorations [60]. Biochemical challenges, such as biofilm accumulation and temperature fluctuations, create harsh environmental conditions for the tooth structure and adhesive interfaces. These challenges can lead to processes like hydrolysis, leaching, fatigue, and cracking [74]. Dietary habits play a crucial role in maintaining or disturbing the homeostasis of the oral environment. Frequent exposure to sugars and fermentable carbohydrates leads to prolonged plaque acidification [36], resulting in microbiota metabolic activity and tooth demineralization. This acidification can severely damage composite restoration surfaces [37]. Patients are advised to use both a toothbrush and dental floss to remove plaque and food debris from dental surfaces, with dental floss being particularly effective for accessing areas that are challenging to reach with a toothbrush [29,30]. Additionally, the use of toothpaste, mouthwash, and gels containing fluoride has been extensively validated as effective measures for preventing dental caries. By employing these strategies, patients can maintain the health of their remaining tooth structure [31,32,33] that has been restored and effectively prevent recurrent and secondary caries, which are among the most significant factors contributing to restoration failure.

Salivary fluid is essential for the formation of the oral microbiome and biofilm [48]. It has been demonstrated that proteins secreted into saliva within the oral cavity are valuable for testing and diagnosing dental caries, underlining the significance of saliva in both the progression and detection of this condition [75]. Salivary proteins, such as proline-rich proteins, mucins, histatins, cystatins, and statherins, protect the tooth surface by attracting calcium ions and promoting remineralization. The pellicle they form slows demineralization and reduces microbial adherence, shielding the tooth surface from pH fluctuations. This also enhances the buffering action, leading to acid neutralization from bacterial or non-bacterial sources [76]. Furthermore, they positively influence the reorganization of infected or affected carious dentin. A significant reduction in bacterial counts observed after cavity sealing highlights the antimicrobial activity of salivary proteins [77,78,79]. Salivary proteins include powerful enzymes that naturally inhibit bacterial growth by penetrating bacterial cell walls and neutralizing their pathogenicity. These enzymes also exhibit antifungal and antiviral properties [80]. Various studies have found that the texture of saliva is a crucial factor as well. Saliva that is thick, sticky, and frothy, particularly when it exhibits increased viscosity, has been associated with a higher susceptibility to dental caries [49,81,82,83]. These findings indicate that optimal oral health and a lower incidence of dental caries are associated with normal hydration levels and higher salivary flow rates, pH, and buffering capacity.

Xerostomia, characterized by a reduced quality and quantity of saliva, can elevate the prevalence of caries, and it is more common in female individuals over 50 years old [38,39]. Patients with xerostomia often experience decreased survival rates of dental restorations, particularly those with large composite restorations [84]. Under normal conditions, saliva is oversaturated with calcium hydroxyapatite, which helps prevent dental demineralization. Diseases that affect the quality and quantity of saliva, such as Sjogren’s syndrome, viral infections (HIV and HCV), uncontrolled diabetes, Alzheimer’s disease, hypertension, depression, and cancer, can impact the health of intraoral soft and hard tissues, leading to dental caries and gingivitis [40]. Burning Mouth Syndrome (BMS), associated with bruxism and considered an oral psychosomatic condition, often aligns with salivary gland dysfunction [85]. In patients with BMS, xerostomia is frequently reported as an oral symptom [86]. Practicing good oral hygiene, along with using saliva substitutes to lubricate and protect both soft and hard oral tissues, is essential to help prevent further complications. Bulimia nervosa, characterized by self-induced vomiting and binge eating, has also been linked to altered salivary function. Studies indicate that both resting and stimulated salivary flow rates are reduced in patients with sialadenosis, while the total protein and amylase levels are increased [40]. Additionally, xerostomia is associated with over 500 medications, with polypharmacy being the most prevalent cause [41]. Many patients on xerogenic medications are unaware of their risk for oral complications such as dental caries. Medications that contribute to xerostomia include antipsychotics, antihistamines, antiemetics, antiretroviral therapy, decongestants, appetite suppressants, and diuretics [40]. Chemotherapy and radiotherapy of the head and neck can also lead to changes in saliva quality and quantity [40]. It has been verified that while direct damage to teeth or restorations from radiation itself may not be the primary cause of early restoration failure in radiation-related caries, changes in saliva quantity and quality, challenges in maintaining oral hygiene, and an increased cariogenic diet may play more significant roles [38,39]. Furthermore, several studies have linked smoke exposure to an increased risk of caries, primarily due to changes in saliva. [43,44]. Research shows that smokers experience reduced saliva flow [87], and there is a direct connection between nicotine use and elevated levels of cariogenic bacteria, such as *Streptococcus mutans* and *Lactobacillus* [88,89,90]. Consequently, it is crucial to educate patients about the relationship between tobacco smoking and the heightened risk of caries.

The use of caries risk assessment (CRA) can help identify risk factors and is crucial for tailoring patient treatment, as it is directly related to the rate of dental restoration failure [45,50]. Patients with a high or medium caries risk have a two to three times higher risk of restoration failure [14]. A study found that patients with a high DMFT index (decayed, missing, and filled Teeth) have a 2.45 to 4.40 times greater risk of restoration failure compared to those with a low index [61,91]. Additionally, the absence of teeth and inadequate proximal contacts have also been related to lower survival rates for restorations [53].

Missing teeth may lead to the use of partial removable dentures, which can compromise the survival of restorations, as patients with removable dentures frequently struggle to clean them effectively [92,93]. In the elderly population, particularly among those with dementia, epilepsy, those who have suffered a stroke, individuals with low income, or who are uninsured, the improper cleaning of partial dentures is more common [94,95]. Research indicates that at least 40% of senior patients fail to disinfect their dentures adequately [96]. Ulcerations of the gums and/or oral mucosa have been reported in these patients due to a poor denture fit and improper use, further complicating their oral hygiene compliance [97]. Additionally, the presence of non-oral pathogenic bacteria and significant biofilm formation in the oral cavities of patients using dentures have been shown to increase the risk of systemic infections and complicate oral hygiene management [98]. Therefore, professional guidance and proper education on denture care are crucial for these individuals, especially the elderly, patients with special needs, and their caregivers [99].

A compromised periodontal status has also been linked to a higher likelihood of restoration failure, as optimal restoration success is achieved when the surrounding tissues are healthy and stable [100]. Poor periodontal health can alter the oral environment by reducing salivary flow and changing the pH levels, which, along with gingival recession exposing vulnerable root surfaces, increases the risk of caries. Additionally, periodontal disease can complicate caries treatment, particularly when deep pockets or gingival inflammation are present [100,101]. Eliminating periodontal disease before, during, and after restorative procedures is crucial for the longevity of restorations [102].

Psychological factors also play a significant role in enhancing restoration survival [93,94]. Caries risk may increase across age groups due to physiological changes, often linked to increased medication use and diminished self-care. Additionally, drug abuse has been associated with numerous physical and mental health problems, including severe oral health issues and depression [103,104]. These complications often stem from neglected self-care, a common behavior among addicts due to lifestyles characterized by risk-taking, poor oral hygiene, aggression, and carelessness [105,106]. Addicts frequently overlook their health issues, seeking only emergency treatment [107,108]. Oral health problems associated with drug abuse—including the use of opiates, cannabis, hallucinogens, cocaine, and amphetamines—include generalized tooth decay, particularly on smooth and cervical surfaces, salivary hypofunction leading to xerostomia, burning mouth sensation, and decreased saliva pH, all of which heighten the risk of dental caries [109,110,111]. A study on heroin users found that these patients suffer from progressive dental caries regardless of their oral hygiene practices [112]. Erosion is also a frequent issue among drug users, leading to dental wear, which is a risk factor for restorative treatment if it remains uncontrolled [113]. Patients with bulimia have shown an elevated rate of restoration failure due to erosion [73]. Additionally, stress factors can manifest in behaviors such as parafunctional habits, including bruxism, which can induce occlusal stress and pose mechanical challenges to both restorations and adhesive interfaces, potentially resulting in wear and/or cracking of teeth and restorations [67]. Consequently, psychological disorders and drug abuse can adversely affect the longevity of dental restorations through drug side effects or destructive behaviors. Therefore, a tailored approach is needed to treat and follow-up with these individuals.

Several studies have shown the correlation between age and restoration failure. Increased failure rates for direct resin composite restorations were observed during adolescence and after age 65 [60,74,114]. While adolescents are more prone to frequent sugary snacks and soft drinks [13], older patients are more likely to have older restorations, and caries incidence is higher due to changes in their stomatognathic system, impaired motor function, reduced salivary flow, changes to softer diets, general health issues, increased use of medications, and their inability to maintain their oral hygiene [115].

Regarding gender, some studies have reported lower restoration survival in male patients [60,116], while others did not find any correlation [34]. This discrepancy may be due to men typically having stronger bite forces than women, leading to higher rates of material fatigue, fractures, and restoration failures [54]. Additionally, women, being more health-conscious, tend to attend dental hygiene consultations more regularly, which contributes to better oral health outcomes [51,117].

Socioeconomic deprivation has been reported as an important factor in the survival rate of composite restorations [45,50]. Cultural, educational, sociological, and psychological factors also play crucial roles in influencing oral care and, consequently, the longevity of dental restorations [46,47,118,119]. People from poorer socioeconomic strata experience more restoration failures than those from wealthier backgrounds. Restorations in clinics in deprived areas have higher annual failure rates (5.6%) compared to areas with a medium (4.2%) and high (5.1%) socioeconomic status [120]. The educational level of patients and their access to oral health services can affect clinical outcomes [116]. Poor adherence to oral health advice [93], irregular dental visits [121], and lower maternal education at childbirth [52] are other socioeconomic-related factors strongly associated with restoration failure. Conversely, studies have identified a direct correlation between the frequency of dental checkups per year and the failure rates of direct restorations [114]. Records from the General Dental Services of England and Wales indicate that patients who frequently visit dental practices experience significantly reduced survival rates of direct restorations [122,123]. This suggests that a proactive or aggressive approach by dentists may potentially lead to over-treatment [124,125]. This phenomenon can also be explained by the method of determining recall intervals, which takes into account patient-related factors such as oral hygiene, dental history, and age. As a result, patients with poorer indicators are scheduled for shorter intervals between checkups.

Furthermore, research has shown that patients who frequently change dentists are more likely to have their restorations replaced. Dentists who did not place the original restoration are more inclined to replace it than those who did [63,64,65,126,127]. This tendency may stem from dentists having greater trust in their own work than in the work of their peers. Additionally, when addressing a defective restoration in a new patient, dentists lacking baseline information for an accurate prognosis are more likely to replace the entire restoration rather than repair it [66].

### 4.2. Operator-Related Factors

The operator’s technique plays a pivotal role in enhancing the longevity of dental restorations [13,68,128,129]. Proper moisture control and meticulous adherence to restorative protocols are crucial during the execution of technique-sensitive steps in adhesive dentistry [20]. Additional considerations include employing the correct light-curing technique, which depends on factors such as the type of light-curing unit (LCU), the duration of light exposure, the thickness of the resin composite, and positioning the light-curing tip as close to the work area as possible. For surfaces that are difficult to reach, extending the light-curing time is recommended [56,57,120,130,131,132,133,134,135,136,137]. It is crucial for clinicians to understand and optimize the photopolymerization process of resin-based composites to enhance material properties. A previous study by Kopperud et al. (2017) revealed that the majority of Norwegian dental practitioners lacked adequate knowledge in this area. Additionally, a significant portion of the participants (78.3%) were not aware of the irradiance values of their light-curing units (LCUs). The study also found that the same percentage of dentists neglected regular maintenance of their LCUs [58]. Composite restorations require adequate radiant exposure to properly polymerize, ensuring optimal mechanical and physical properties [59]. Insufficient photopolymerization may lead to color changes over time [138,139,140], which could mistakenly be interpreted as recurrent caries. Additionally, inadequate polymerization is linked to lower microhardness values [141], the degradation of the restoration, the weakening of its mechanical properties, and an increased risk of microleakage [142].

An interesting observation is the higher annual failure rates of dental restorations placed in large group practices compared to those performed by a single dentist or in smaller practices [60]. This finding may be related to the fact that patients in larger practices are often seen by different dentists. It has been previously observed that changing dentists leads to a higher rate of restoration replacements [122,123]. Additionally, long-term studies on posterior composites placed by general practitioners indicate that their survival rates are comparable to those placed by specialists [143].

The dentist’s decision-making process is another critical factor that can significantly impact the longevity of restorations [14,144]. This process is often underestimated and involves various complex co-variables [120]. The presence of additional risk factors, such as tooth vitality [70], the number of remaining walls [35,71], and the use of removable partial dentures [145], further complicates the decision-making process. Moreover, securing the patient’s consent for proposed treatment options adds another layer of complexity in selecting the most suitable treatment when planning the restoration of a defective tooth [11,13,146,147,148,149,150,151,152,153,154,155]. A potential explanation for the wide variation in annual failure rates (AFRs) observed for composite restorations relates to whether dentists adopt a proactive or reactive approach to aged restorations. Proactive dentists, who prefer to intervene early rather than monitor a questionable restoration, may contribute to shorter restoration longevity compared to reactive, more conservative dentists who wait to observe disease progression before intervening. Additionally, a proactive approach does not necessarily guarantee that the new restoration will outlast the original [120]. Success and survival rates tend to decrease when dentists choose to repair or replace restorations due to minor issues such as marginal staining, gaps, chipping, or wear [114]. In a retrospective study spanning up to 18.5 years, restorations that underwent more frequent annual checkups exhibited higher failure rates than those with fewer checkups. The variability in dentists evaluating the restorations significantly affected the time until failure [114]. While some studies have verified the influence of the operator on survival rates, the dentist’s impact on the outcomes is not always clear [122,123,144].

Regarding the operator’s age, studies present conflicting findings about the impact of a dentist’s experience and age on the survival of dental restorations. Some studies indicate that restorations performed by less experienced dentists or those who graduated more recently have higher annual failure rates [74]. Conversely, other studies suggest that restorations placed by younger dentists tend to last longer [156]. Additionally, one study found that restorations performed by dentists who graduated within the past 19 years are less likely to fail compared to those performed by dentists who graduated over 19 years ago [143]. This study showed that the time from initial restoration placement to the need for reintervention is shorter for restorations placed by older dentists [122]. The evolution of materials and techniques may explain this fact, as older dentists may not always be up-to-date with the most recent materials and restorative protocols.

Regarding the dentist’s gender, a study found that restorations placed by male clinicians were less likely to fail compared to those placed by female clinicians [156]. This may be related to another finding that part-time practitioners have higher failure rates than full-time practitioners, with a higher proportion of women working in part-time positions compared to men [157,158,159,160]. The differences identified in multivariable analyses could also stem from varying diagnosis patterns; for instance, women might be more likely to report a restoration as a failure, whereas men might not.

It has also been shown that dentists’ motivation and workload potentially influence their treatment outcomes [122]. Practice workload emerged as a significant predictor, though the reasons behind this observation remain unclear. Interestingly, dentists reporting a need for more patients tend to have a success rate approximately twice that of balanced or slightly busy clinicians. One might speculate that a dentist with sufficient time could dedicate more attention to detail and take longer with a procedure. However, clinicians describing their practice as excessively busy may also exhibit a high success rate, possibly indicating greater efficiency. Practices categorized as very busy or not busy demonstrated restorations with higher survival rates, suggesting a complex interplay of practice style, work environment, and clinical approach [115,161].

### 4.3. Tooth-Related Factors

Teeth with larger cavities or fewer cavity walls, especially when marginal ridges are removed, present increased cuspal deflection during function [62]. This leads to a higher likelihood of fractures and an increased annual failure rate (AFR) [69,162]. Teeth with class I restorations rarely fracture due to excessive loads, as the remaining walls and the presence of marginal ridges—considered reinforcing structures by connecting facial and lingual walls—provide stability. In class II restorations, the wedge effect combined with the load remains within the cavity, causing additional stresses that may lead to fractures in the cavity walls [163]. The width of the isthmus has a significant impact on class II preparations. An isthmus measuring one-fourth of the intercuspal distance can provide increased strength to the prepared tooth [69]. Marginal ridges reinforce the tooth by connecting the facial and lingual walls. MOD cavities, which involve a significant loss of dentin, are considered the most prone to fractures and have a higher annual failure rate (AFR) [164].

Regardless of the treatment used, studies suggest a strong relationship between the amount of remaining tooth tissue and restorations’ survival [16,165,166]. Opdam (2014) reported that the risk of AFR increased by 30% to 40% for each additional missing wall. Furthermore, resin composite restorations failed 3.3 times more often in posterior teeth with fewer than two remaining walls compared to those with four walls [55]. Additionally, endodontically treated molars with restorations covering more than four surfaces exhibited higher failure rates [10]. Studies show that the reasons for the failure of composite restorations vary depending on the time of evaluation. Failures during the follow-up period of one to four years are mostly related to restoration fractures. In contrast, secondary caries and the need for endodontic treatment become the predominant causes of failure over a ten-year evaluation period [166,167].

The quality of the remaining tooth structure is another crucial factor for enhancing restoration longevity. Factors such as the presence of cracks, sclerotic and deep dentin compared to sound and superficial dentin, and the position of cervical margins are important aspects influencing the survival rate of restorations [27,168,169]. Furthermore, restorations placed due to fractures were more prone to fail than those placed due to caries [74]. Additionally, the presence of deep caries lesions showed a four times higher risk of failure, while cavities with pulp exposure had a fourteen times higher failure risk compared to restorations localized in the dentin [170].

Relating to tooth vitality, the need for endodontic treatment is a critical factor influencing the prognosis, long-term survival, and success of teeth and restorations. A practice-based study with over 430,000 restorations reported that fewer than 20% of restorations placed in endodontically treated teeth survived after 10 years. The main reasons for failures included secondary caries, root and cusp fractures, loss of retention, and restoration fractures. In vital teeth, composite restorations mainly fail due to secondary caries and restoration fractures [67,116,156]. Endodontic treatment has been shown to reduce the stiffness of teeth by 5%, while an occlusal restoration can reduce stiffness by 20%, and a MOD restoration can reduce tooth stiffness by 63% [42]. A previous study reported that the annual failure rate (AFR) for endodontically treated teeth was 10.9%, compared to 4.5% for vital teeth [60]. Root-filled teeth undergo a reduction in free water content within the dentine matrix and tubules, affecting their viscoelastic properties. Dehydration is suggested as a contributing factor to vertical root fractures. The most critical factor is the loss of tooth structure, and there is general consensus that the cuspal coverage of endodontically treated posterior teeth enhances their survival [72].

Tooth position and antagonistic contacts can also play a significant role in the longevity of restorations [53]. The superior visibility of occlusal tooth surfaces facilitates an easier and earlier detection of secondary caries and other restoration deficiencies [171]. Molars generally have lower restoration survival rates than other teeth due to larger restorations and greater occlusal forces [115]. The first molar, the most frequently restored tooth, showed the highest AFR [74]. Additionally, studies indicated that restorations might last longer in premolars than in molars [14]. Restorations in the upper arch had a higher risk of failure compared to those in the lower arch. A study found that after 5 years, direct composite restorations exhibited more wear in the upper arch than in the lower arch, though the reason for this remained unclear [12]. Contrary to these findings, Lucarotti (2018) reported that the restoration survival rate over 15 years varies by tooth type, with upper molars having the highest survival rate. Another study revealed that the AFR of restorations was similar across the four quadrants [74].

This study’s limitations stem from its interdisciplinary scope. While it focuses on the three main factors influencing restoration longevity—patient, tooth, and operator-related factors—each involves diverse research areas. Tooth-related factors are usually examined in clinical trials assessing materials and techniques, while patient-related factors are broader, covering oral hygiene, diet, and the effects of certain diseases, smoking, alcohol, and drug use on cariogenic bacteria and saliva flow. Psychological and psychomotor performance also play a role. However, prospective trials often exclude patients with “non-ideal” conditions, limiting the assessment of different patient scenarios. Most studies on medical conditions focus on surgical outcomes rather than restoration longevity. While the operator’s role is well-documented in the literature, more emphasis is needed on their responsibility in educating patients about risk factors.

In conclusion, this review underscores the need for a holistic approach in dental practice, where understanding and addressing multifactorial contributors can lead to improved restoration success rates and reduced failure rates. Despite advancements in restorative materials, failure rates remain significant, leading to costly replacements and additional dental trauma. Continuous education and training for dental professionals, coupled with comprehensive patient management, are recommended to optimize the longevity of dental restorations. By addressing patient-related factors such as diet, oral hygiene, systemic health conditions, and socioeconomic status; dentist-related factors like technique and applied knowledge; and tooth-related factors to tailor the most suitable restorative treatment for each individual case, dental professionals can significantly improve patient outcomes, reduce the need for frequent replacements, and ultimately enhance the overall quality of dental care. Several studies have emphasized the need for tailored treatment based on individual patient factors. Furthermore, a more conservative approach with an emphasis on prevention has been the ultimate goal. The findings in this review indicate that the survival of direct restorations is not solely dependent on tooth-related factors, but it is also significantly influenced by the complex interplay of several factors. Investing in research and development for advanced materials and techniques, enhancing training programs for dental practitioners with a focus on patient education, developing robust patient education initiatives, and fostering collaboration between dental and medical professionals are crucial steps. By addressing these multifactorial contributors through a comprehensive and informed approach, dental professionals can significantly improve dental restoration outcomes, benefiting both patients and healthcare systems.

## Figures and Tables

**Table 1:** Patient-related factors influencing the longevity of dental restorations.

Patient-Related Factors	Description	References
Oral Hygiene Practices	Type of toothpaste used, frequency of brushing, flossing habits, and use of mouthwash.	Heintze et al., 2012 [9]; De Paola, 1983 [29]; Murray, 1991 [30]; Marinho, 2003, 2004 [31,32]; Twetman, 2004 [33]; Van de Sande et al., 2013 [34]; and Opdam et al., 2010 [35].
Dietary Habits	Frequency of snacking, consumption of fermentable carbohydrates, and acid exposure from food and drink.	Chapple et al., 2017 [17]; Pitts et al., 2021 [25]; Marsh, 2003 [36]; and Beyth et al., 2008 [37].
Medical Conditions	Systemic diseases (e.g., diabetes and xerostomia) and the use of medications causing dry mouth.	Nederfors et al., 1997 [38]; Villa et al., 2011 [39]; Iorgulescu, 2009 [40]; Scully, 2003 [41]; and Reeh et al., 1989 [42].
Lifestyle Factors	Smoking and parafunctional habits (e.g., bruxism).	Benedetti et al., 2013 [43]; Jiang et al., 2019 [44]; and Thomson et al., 2004 [45].
Psychological Factors	Stress and associated behaviors (e.g., bruxism) and psychological conditions affecting self-care.	Bauer et al., 2012 [46] and Witt and Flores-Mir, 2011 [47]
Salivary Factors	Salivary flow rate and composition and the presence of salivary proteins and enzymes.	Proctor, 2016 [48]; Kaur et al., 2012 [49]; and Nederfors et al., 1997 [38].
Socioeconomic Status	Access to dental care, education level, and adherence to oral health advice.	Peres et al., 2007 [50]; Thomson et al., 2004 [45]; Crocombe et al., 2012 [51]; and Correa et al., 2013 [52].
Age and Gender	Age-related changes in oral health and gender differences in oral health practices.	Linnemann et al., 2021 [53]; Silva et al., 2015 [54]; and Ayers et al., 2008 [55].

**Table 2:** Summary of dentist-related factors affecting the survival of dental restorations.

Operator-Related Factors	Description	References
Experience and Technique	The longevity of restorations was positively correlated with the dentist’s experience, particularly in adhesive protocols and moisture control techniques. Inadequate curing and poor isolation during procedures were major contributors to restoration failure.	Ferracane and Lawson, 2021 [56]; Jandt and Mills, 2013 [57]; Kopperud et al., 2017 [58]; Maktabi et al., 2018 [59]; and Opdam et al., 2010 [35].
Practice Environment	Restorations placed in larger group practices exhibited higher failure rates than those placed in smaller practices, potentially due to variations in technique and patient management among different practitioners.	Laske et al., 2016 [60]; Trachtenberg et al., 2008 [61]; Linnemann et al., 2021 [53]; Van de Sande et al., 2013 [34]; and Collares et al., 2017 [62].
Decision-Making Process	Dentists’ approach to treatment—whether proactive (early intervention) or reactive (monitoring and delayed intervention)—impacted the longevity of restorations, with proactive approaches sometimes leading to over-treatment and higher failure rates.	Lucarotti and Burke, 2018 [22]; Elderton, 1977 [63]; Elderton and Nuttall, 1983 [64]; Bader and Shugars, 1992 [65]; and Gordan et al., 2012 [66].

**Table 3:** Key tooth-related factors impacting the longevity of dental restorations.

Tooth-Related Factors	Description	References
Cavity Size and Location	Larger cavities and those located in posterior teeth, particularly molars, exhibited higher failure rates. MOD cavities and those involving significant dentin loss were more prone to fractures.	Opdam et al., 2014 [16]; Lempel et al., 2019 [67]; Van Nieuwenhuysen et al., 2003 [68]; and Mondelli et al., 1980 [69].
Tooth Vitality	Restorations on endodontically treated teeth had a higher failure rate compared to vital teeth. Teeth requiring endodontic treatment were at a higher risk of restoration failure due to reduced tooth structure and changes in biomechanical properties.	Lempel et al., 2019 [67]; Mogano et al., 2004 [70]; Trushkosky, 2014 [71]; Reeh et al., 1989 [42]; and Bhuva et al., 2021 [72].
Tooth Position	Restorations on molars had a lower survival rate than those on premolars or anterior teeth, likely due to higher occlusal forces.	Opdam et al., 2014 [16]; Lucarotti and Burke, 2018 [22]; Bartlett and Sundaram, 2006 [73]; and Van Nieuwenhuysen et al., 2003 [68].

## Data Availability

Data will be available by request to the correspondent author.
